# Multidimensional Analysis of the Adult Human Heart in Health and Disease Using Hierarchical Phase-Contrast Tomography

**DOI:** 10.1148/radiol.232731

**Published:** 2024-07-16

**Authors:** Joseph Brunet, Andrew C. Cook, Claire L. Walsh, James Cranley, Paul Tafforeau, Klaus Engel, Owen Arthurs, Camille Berruyer, Emer Burke O’Leary, Alexandre Bellier, Ryo Torii, Christopher Werlein, Danny D. Jonigk, Maximilian Ackermann, Kathleen Dollman, Peter D. Lee

**Affiliations:** From the Department of Mechanical Engineering, University College London, London, England (J.B., C.L.W., C.B., E.B.O.L., R.T., P.D.L.); European Synchrotron Radiation Facility, 71 Av des Martyrs, 38000 Grenoble, France (J.B., P.T., C.B., K.D.); UCL Institute of Cardiovascular Science, London, England (A.C.C.); Wellcome Sanger Institute, Hinxton, England (J.C.); Siemenst Healthineers, Erlangen, Germany (K.E.); Department of Radiology, Great Ormond Street Hospital for Children NHS Foundation Trust, London, England (O.A.); Laboratoire d’Anatomie des Alpes Françaises, Université Grenoble Alpes, Grenoble, France (A.B.); Institute of Pathology, Hannover Medical School, Hannover, Germany (C.W.); Biomedical Research in Endstage and Obstructive Lung Disease Hannover, German Center for Lung Research (DZL), Hannover, Germany (D.D.J.); Institute of Pathology, Faculty of Medicine, RWTH Aachen University, Aachen, Germany (D.D.J., M.A.); Institute of Pathology and Molecular Pathology, Helios University Clinic Wuppertal, Universität Witten/Herdecke, Wuppertal, Germany (M.A.); Institute of Functional and Clinical Anatomy, University Medical Center of the Johannes Gutenberg–University Mainz, Mainz, Germany (M.A.); and Research Complex at Harwell, Didcot, England (P.D.L.).

## Abstract

**Background:**

Current clinical imaging modalities such as CT and MRI provide resolution adequate to diagnose cardiovascular diseases but cannot depict detailed structural features in the heart across length scales. Hierarchical phase-contrast tomography (HiP-CT) uses fourth-generation synchrotron sources with improved x-ray brilliance and high energies to provide micron-resolution imaging of intact adult organs with unprecedented detail.

**Purpose:**

To evaluate the capability of HiP-CT to depict the macro- to microanatomy of structurally normal and abnormal adult human hearts ex vivo.

**Materials and Methods:**

Between February 2021 and September 2023, two adult human donor hearts were obtained, fixed in formalin, and prepared using a mixture of crushed agar in a 70% ethanol solution. One heart was from a 63-year-old White male without known cardiac disease, and the other was from an 87-year-old White female with a history of multiple known cardiovascular pathologies including ischemic heart disease, hypertension, and atrial fibrillation. Nondestructive ex vivo imaging of these hearts without exogenous contrast agent was performed using HiP-CT at the European Synchrotron Radiation Facility.

**Results:**

HiP-CT demonstrated the capacity for high-spatial-resolution, multiscale cardiac imaging ex vivo, revealing histologic-level detail of the myocardium, valves, coronary arteries, and cardiac conduction system across length scales. Virtual sectioning of the cardiac conduction system provided information on fatty infiltration, vascular supply, and pathways between the cardiac nodes and adjacent structures. HiP-CT achieved resolutions ranging from gross (isotropic voxels of approximately 20 µm) to microscopic (approximately 6.4-µm voxel size) to cellular (approximately 2.3-µm voxel size) in scale. The potential for quantitative assessment of features in health and disease was demonstrated.

**Conclusion:**

HiP-CT provided high-spatial-resolution, three-dimensional images of structurally normal and diseased ex vivo adult human hearts. Whole-heart image volumes were obtained with isotropic voxels of approximately 20 µm, and local regions of interest were obtained with resolution down to 2.3–6.4 µm without the need for sectioning, destructive techniques, or exogenous contrast agents.

© The Author(s) 2024. Published by the Radiological Society of North America under a CC BY 4.0 license.

*Supplemental material is available for this article.*

See also the editorial by Bluemke and Pourmorteza in this issue.

## Graphical abstract

**Figure:**
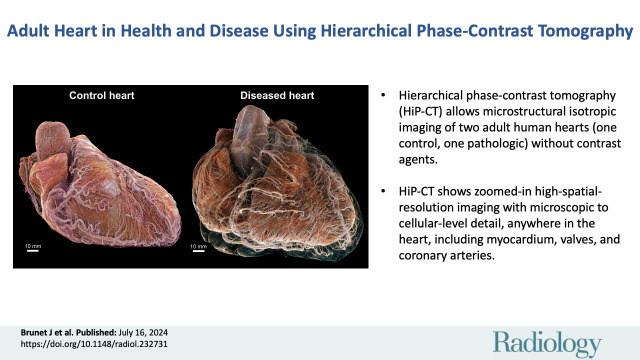

**Summary**

Hierarchical phase-contrast tomography (HiP-CT) enables ex vivo virtual autopsy cardiac imaging with high spatial resolution, providing nondestructive, three-dimensional, multiscale analysis of intact healthy and diseased adult human hearts without contrast agents.

**Key Results**

■ Hierarchical phase-contrast tomography (HiP-CT) enabled nondestructive, microstructural, whole-heart imaging of two adult human hearts (one control and one pathologic) with isotropic voxels of approximately 20 µm without contrast agents.
■ HiP-CT allowed zoomed-in high-spatial-resolution imaging with microscopic (approximately 6.5-µm voxel size) to cellular-level (approximately 2.3-µm voxel size) detail, anywhere within the heart—including the myocardium, valves, and coronary arteries—without tissue sectioning.
■ Virtual multiplanar sectioning via HiP-CT of the cardiac conduction system revealed insights into the nodal areas, vascular supply, and surrounding structures.

## Introduction

A comprehensive understanding of the structurally normal and diseased human heart is crucial to improve understanding of pathophysiologic processes and therefore diagnosis and treatment of cardiovascular diseases.

Synchrotron x-ray phase-contrast imaging has been used to image whole hearts but is limited in size to human fetal and small animal hearts. For these tiny hearts, image resolution down to 5.2 µm (1) has been achieved using x-ray phase-contrast imaging, with regions of interest depicted with resolution down to 0.65 µm (2). This is orders of magnitude better than current clinical imaging techniques such as CT (3–5) and does not require invasive dissection (6) but has been limited in size to a few centimeters. Other techniques—such as light-sheet fluorescence microscopy (7,8), laboratory micro-CT, and nanotomography (9–12)—require exogeneous contrast agents or are limited in field of view and to small sample. Three-dimensional (3D) diffusion-tensor MRI has promise for allowing inference of myocardial architecture, providing 500-µm voxel resolution in adult atria, but is unable to depict structure directly due to limited resolution (13). All of these limitations can potentially be addressed using x-ray phase-contrast imaging, as the brightness (x-ray intensity) of synchrotron sources eliminates the need for contrast agents and provides sufficient resolution to resolve cardiac microstructure directly (12). Nonetheless, imaging larger adult human hearts at high spatial resolution, while overcoming field-of-view and resolution limitations, has been challenging.

Recently, an x-ray phase-contrast imaging technique, hierarchical phase-contrast tomography (HiP-CT), was developed that is capable of imaging intact adult human organs with cellular resolution (14) using a synchrotron source at the European Synchrotron Radiation Facility, the Extremely Brilliant Source. This latest fourth-generation synchrotron advancement offers substantial improvements over previous machines in x-ray brilliance and high energies, enabling large organs to be rapidly scanned with high contrast and high spatial resolution (15).

Phase-contrast imaging differs from conventional x-ray imaging, which relies on x-ray absorption, in exploiting the phase shift (or refraction) of x-rays passing through tissues, rather than relying solely on x-ray absorption. It results in images with dramatically higher contrast and resolution, which is particularly beneficial for visualizing soft tissues and fine structures, as found in the heart, without the need for exogenous contrast agent. This study aimed to evaluate the capability of HiP-CT to depict the macro- to microanatomy of structurally normal and abnormal adult human hearts ex vivo.

## Materials and Methods

### Sample Preparation

In this exploratory study, between February 2021 and September 2023, two hearts were obtained from bodies donated to the Laboratoire d’Anatomie des Alpes Françaises, following French legislation for body donation. Transport and imaging protocols were approved by the United Kingdom Health Research Authority and Integrated Research Application System (200429) and the French Ministry of Health.

The first heart, from a 63-year-old White male, served as the control. No clinically significant cardiovascular disease had been recorded. The second heart was from an 87-year-old White female with a history of multiple cardiac and extracardiac comorbidities including ischemic heart disease, hypertension, and atrial fibrillation (Table).

**Table:**
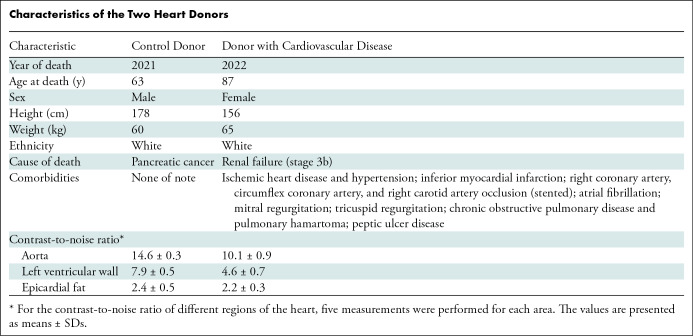
Characteristics of the Two Heart Donors

### HiP-CT Imaging Procedure

Nondestructive ex vivo imaging without exogenous contrast agents was performed using HiP-CT at the European Synchrotron Radiation Facility. Hearts were prepared and imaged using beamline BM18 essentially as described in detail by Walsh et al (14) (Appendix S1). Scanning was performed using a polychromatic parallel beam tuned using a combination of molybdenum attenuators, with an average energy range of 94–126 keV (see Fig S1 for schematic). A hierarchical approach to imaging was employed, starting with HiP-CT of the complete heart (isotropic voxel size of approximately 20 µm), followed by local “zoom” scans in regions of interest (isotropic voxel size of approximately 6.4 µm and approximately 2.3 µm; see Appendix S1). Comprehensive details regarding the scan parameters for the three distinct voxel sizes, including the scan time, number of projections, x-ray beam energy, and dose rate, are provided in Table S1.

### Data Processing and Analysis

Scans were reconstructed using a filtered back-projection algorithm coupled with single-distance phase retrieval (16) and a two-dimensional unsharp mask using the software package PyHST2 version 2022c (European Synchrotron Radiation Facility), a hybrid distributed reconstruction code (17) (*https://software.pan-data.eu/software/74/pyhst2*). Following vertical concatenation of scans, ring artifact correction was performed on the reconstructed sections using an updated Lyckegaard algorithm (18). An overview and details of the reconstruction pipeline, adapted from Xian et al (19), can be found in Figure S2. Data size ranged from 150 to 915 GB per reconstructed volume and required ultra-high-performance computing to process and visualize as described by Brunet et al (20) (Appendix S1). The data from this study are publicly accessible through the web interface Neuroglancer (21) (Appendix S1, Fig S3).

Volumes were manually registered for visualization and semiautomatically segmented using a bounded region growing algorithm in VGSTUDIO MAX 3.5.1 (Volume Graphics). For demonstration of normal heart anatomy and comparison with diseased heart anatomy, data sets were virtually sectioned and reviewed in multiple orthogonal planes. The 3D anatomic renderings were created using Cinematic Anatomy version VB11A (Siemens Healthineers) (22).

### Quantitative Analysis

To show the feasibility of quantitative measurements relevant to clinical cardiology, the thickness of the atrial walls (endocardium, myocardium, and epicardium), orientation of myocyte aggregates in the ventricles, and degree of mitral annular disjunction were calculated. Contrast-to-noise ratio was assessed in different tissue types: aorta, left ventricular wall, and epicardial fat.

### Measurement of Atrial Wall Thickness

Voxel size was predetermined and calibrated during the beamline setup prior to imaging. The thickness of the atrial wall and its constituent sublayers (endocardium, myocardium, and epicardium) were manually determined at 15 evenly distributed locations on calibrated data sets using the measurement function of Fiji software (23). Thickness measurements in the control and diseased hearts, and in the right versus left atrium, were compared. For more information see the Statistical Analysis section.

### Calculation of Myocyte Orientation

The local orientation of myocyte aggregates was determined using the structure tensor method (1). Briefly, intensity gradients were calculated for each voxel, using the Gaussian derivative method, along the three spatial orthogonal directions. Subsequently, the structure tensor was subjected to eigendecomposition, resulting in three eigenvalues and corresponding eigenvectors for each voxel. To identify the primary direction of the myocyte aggregates, the eigenvector associated with the smallest eigenvalue was selected, given that changes in image intensity are not expected to occur along the longitudinal axis of the cardiomyocytes. Finally, the helical angle was computed for each voxel. This angle represents the deviation between the transverse plane and the vector’s projection onto the local tangential plane within the cylindrical coordinate system of the heart. The centerline of the left ventricle was chosen as the center of the cylindrical system.

### Mitral Annular Disjunction Quantification

After finding the centerline of the left ventricle, the degree of displacement of the mitral valve hinge from the adjacent ventricular myocardium was measured in 5° steps around the mural leaflet of the mitral valve in the control heart and the diseased heart.

### Contrast-To-Noise Ratio Quantification

Contrast-to-noise ratio (CNR) was quantified on the full-field images with Fiji using the following formula: 
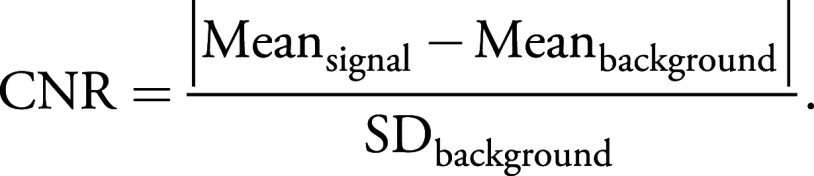


To measure the variation of the CNR in different tissues, quantification was performed in the aorta, left ventricular wall, and epicardial fat. Five measurements were performed for each region. The agar gel was taken as the background. The CNR could not be quantified in zoom scans because no background could be defined. The results are displayed in the Table. These measurements are provided as quick indicators and should be interpreted with caution when comparing them with other imaging techniques, such as clinical CT (24).

### Statistical Analysis

Thickness measurements in the control and diseased hearts and in the right versus left atrium were compared using unpaired multiple *t* tests with multiple comparison correction applied via the Holm-Sidak method and a set α threshold of .05. Analyses were performed using Prism 10.1.2 software (GraphPad).

## Results

**Figure 1:**
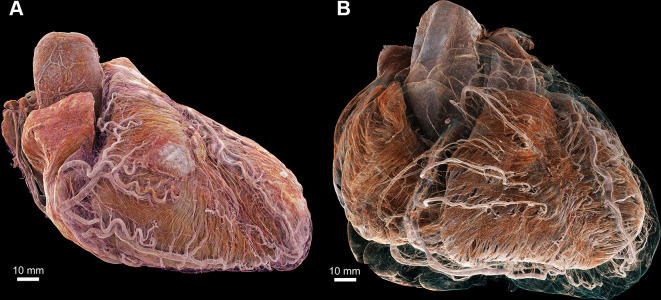
Three-dimensional cinematic renderings of the **(A)** control and **(B)** diseased hearts in anatomic orientation. Epicardial fat has been removed digitally to show the course of the major coronary arteries plus details of smaller arteries penetrating into the myocardium that are not typically seen on clinical CT scans. In the control heart, the coronary arteries remain close to the epicardial surface, while in the diseased heart, they are lifted away by epicardial fat, increasing the perfusion distance between the major coronary arteries and the myocardium. Segmentations and high-spatial-resolution details of coronary arteries in the diseased heart are also shown in Figure 5.

**Figure 2:**
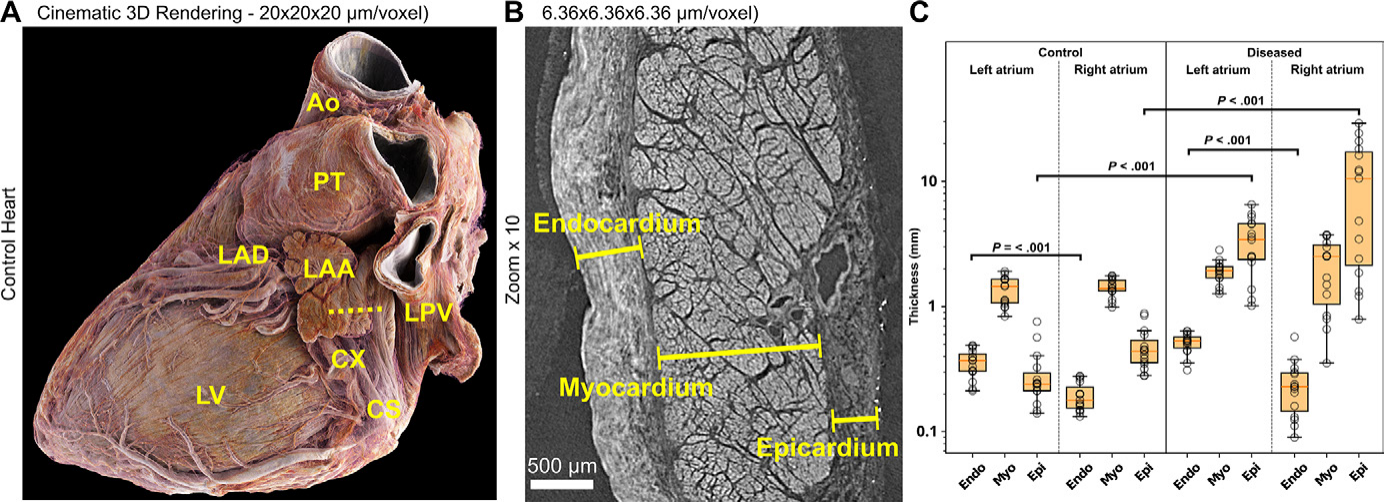
**(A)** Three-dimensional (3D) cinematic rendering from hierarchical phase-contrast tomography (HiP-CT) of the control heart, viewed from the left posterior aspect, with epicardial fat digitally removed using thresholding, shows the left atrium and the left atrial appendage (LAA), which can be seen running over the circumflex coronary artery (CX). This anatomic relationship is a key landmark for interventional closure of the left atrial appendage with devices. Ao = aorta, CS = coronary sinus, LAD = left anterior descending coronary artery, LPV = left pulmonary vein, LV = left ventricle, PT = pulmonary trunk. **(B)** High-spatial-resolution local HiP-CT scan of the wall of the left atrial appendage in the control heart (view plane is given by the dashed line in **A**) shows histologic spatial resolution of endocardial, myocardial, and epicardial layers, allowing measurement of thickness (lines). **(C)** Box and whiskers plots show the thickness of the endocardium (Endo), myocardium (Myo), and epicardium (Epi) in the left and right atria in the control heart and the diseased heart. Circles indicate individual measurements, the boundaries of boxes indicate the lower and upper quartiles, the red lines indicate medians, and the whiskers indicate ranges. Measurements were obtained manually at 15 distinct locations along the left and right atrial walls. Thickness measurements in the control and diseased hearts, and in right versus left atrium, were compared using unpaired multiple *t* tests with multiple comparison correction applied via the Holm-Sidak method and a set α threshold of .05. Statistical analysis revealed significant differences (*P* < .001) in the thickness of the left and right atrial endocardium within the control heart and within the diseased heart and in the thickness of the left and right atrial epicardium between the control heart and the diseased heart.

**Figure 3:**
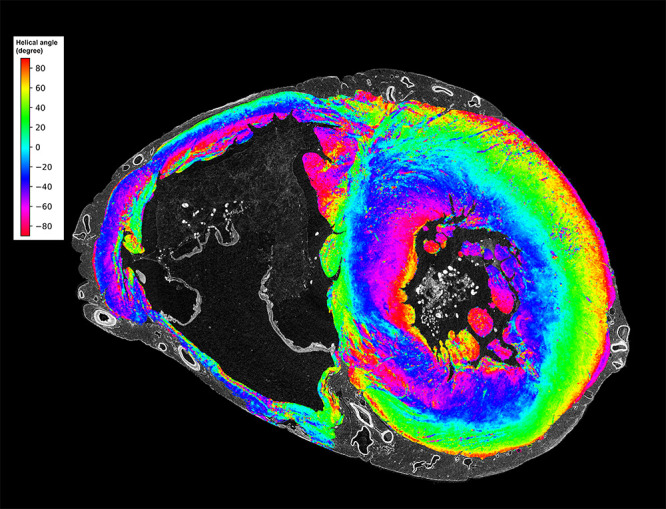
Mid-ventricular short-axis section of the ventricles in the control heart shows the feasibility of myomapping the orientation of myocyte aggregates on hierarchical phase-contrast tomography images at low spatial resolution. Myocyte orientation was calculated by means of structure tensor analysis with an in-house code based on the Python library structure-tensor (5,6). Colors represent the helical angle of aggregates of myocytes, which show a gradual transition in the thicker left ventricle from epicardium to endocardium.

**Figure 4:**
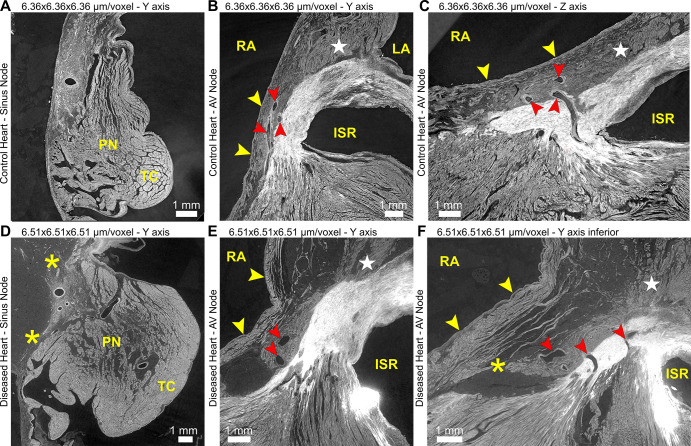
Hierarchical phase-contrast tomography images show the feasibility of identifying and tracing the conduction system with virtual multiplanar sectioning in **(A–C)** control and **(D–F)** diseased heart. In the control heart, the sinus and atrioventricular nodes are tightly adjacent to the surrounding atrial myocardium, paranodal area (PN), and terminal crest (TC), with multiple potential sites of connection. Multiplanar sectioning allows vascular connections from the atrioventricular node to be followed (red arrowheads in **B, C, E, F**). These connections can be seen breaking up the bright fibrous tissue between atrial and ventricular myocardium and represent potential sites of normal nodoventricular connection. In the diseased heart, there is fatty infiltration in the region of the sinus and atrioventricular nodes, separating the respective nodal tissue from surrounding right atrial (RA) myocardium (yellow arrowheads in **B, C, E, F**) and from the paranodal area. This fatty infiltration elongates the connections between nodal tissue and surrounding atrial myocardium (* in **D**). In particular, the connection between the atrioventricular node and the atrial septum (★ in **B, C, E, F**), a potential fast pathway, is diminished in the diseased heart compared with the control heart. An attenuated connection with the vestibule of the right atrium (potential slow pathway) can also be seen in a more inferior section of the diseased heart, made across the inferior pyramidal space (* in **F**). ISR = inferoseptal recess (within the left ventricle), LA = left atrium.

**Figure 5:**
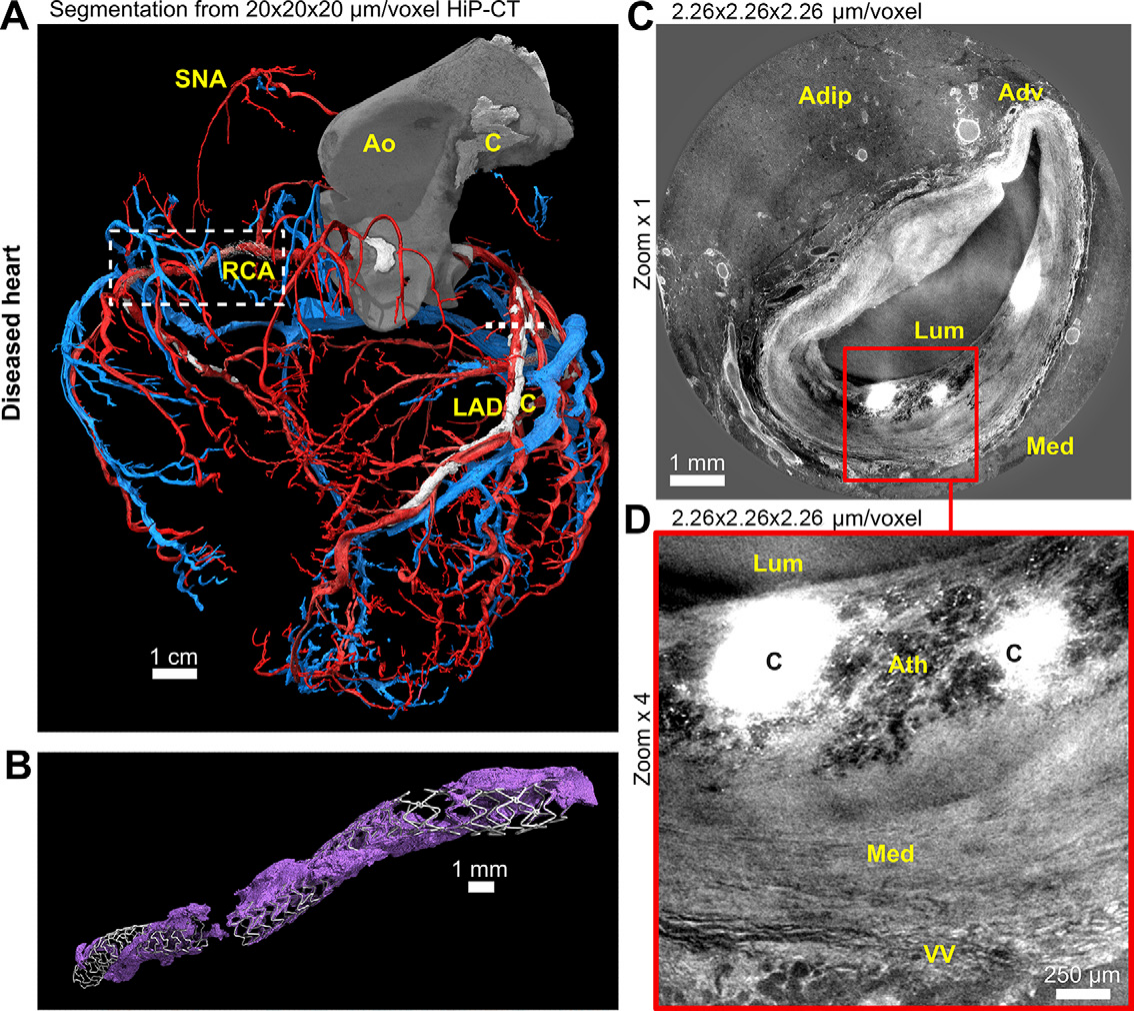
**(A, B)** Three-dimensional cinematic renderings and **(C, D)** images from hierarchical phase-contrast tomography (HiP-CT) show segmentation of the coronary arterial and venous tree in the diseased heart. **(A)** The heart is viewed on its apex to show the course of the major coronary arteries and veins as well as smaller vessels such as the sinoatrial nodal artery (SNA). The coronary vasculature is lifted away from the epicardial surface by lipomatous hypertrophy, lengthening the penetrating arteries (see also Fig 1). Box indicates the location of the stent shown in **B**; line indicates the cross-section shown in **C. (B)** Cinematic rendering of one of the coronary stents, along with surrounding calcification (purple), shows feasibility of imaging both soft and hard tissue with minimal artifacts. **(C)** High-spatial-resolution local tomography of the left descending coronary artery shows atherosclerotic plaque including calcification (box). **(D)** Digital zoom scan of box in **C** shows details of the atherosclerotic plaque and calcification within the intima. Adip = adipose tissue, Adv = tunica adventitia, Ao = aorta, Ath = atherosclerosis, C = calcification, LAD = left anterior descending coronary artery, Lum = lumen, Med = tunica media, RCA = right coronary artery, VV = vasa vasorum.

**Figure 6:**
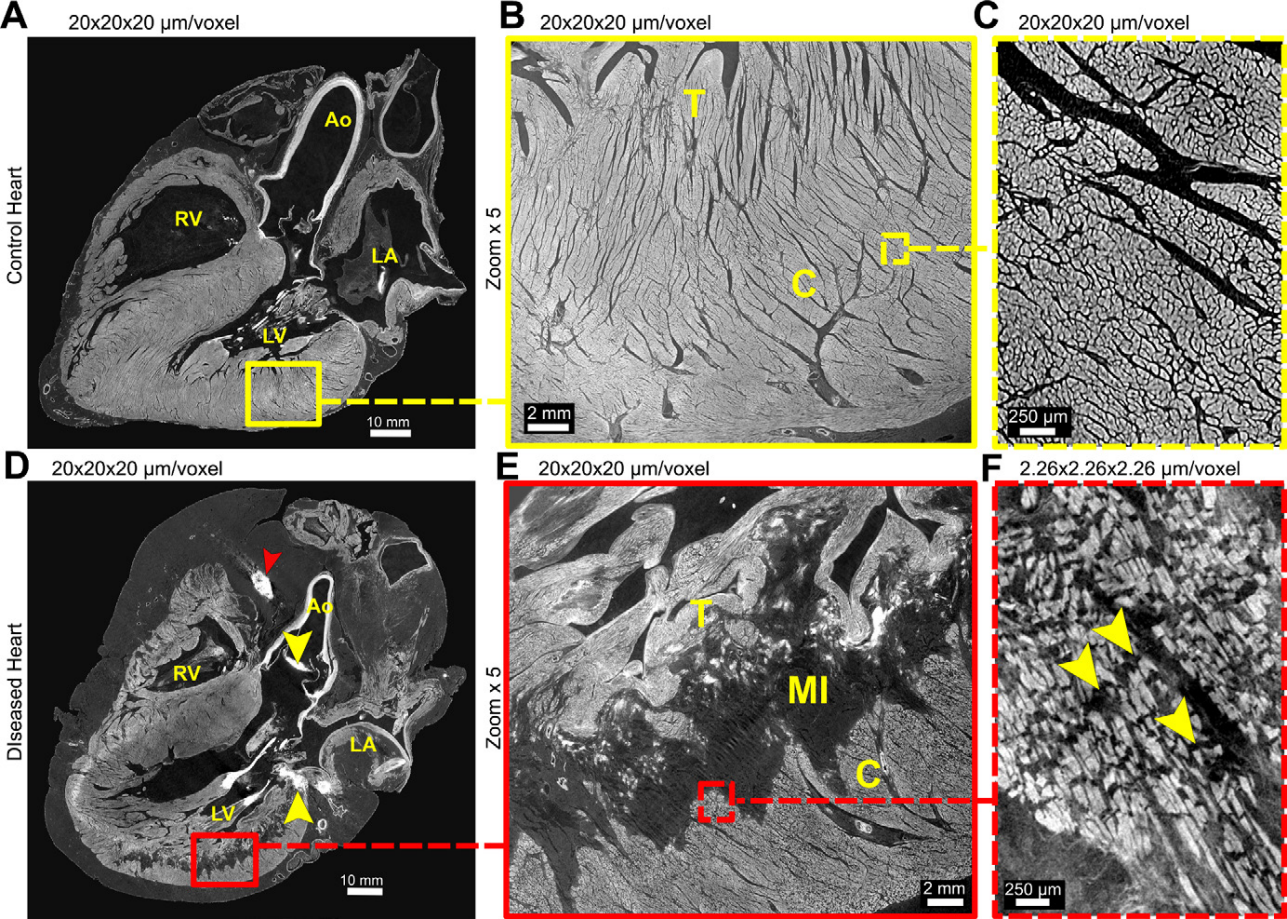
Hierarchical phase-contrast tomography scans of ventricular myocardium in **(A–C)** control and **(D–F)** diseased heart. **(A, D)** Long-axis views show the extent of myocardial infarction (MI) in the diseased heart, together with lipomatous hypertrophy, bright areas of calcification in the aortic valve and mitral annulus (yellow arrowheads in **D**), and stent in the right coronary artery (red arrowhead in **D**). **(B, E)** Zoom scans of boxes in **A** and **D** show the high spatial resolution obtained even at this voxel size and the extensive nature of the myocardial infarction that lies immediately beneath the trabecular myocardium (T). **(C, F)** High-spatial-resolution scans of the boxed areas in **B** and **E** show differences in myocardial aggregates, which are separated by replacement fibrosis in the diseased heart (yellow arrowheads in **F**) but tightly packed in the control heart. Ao = aorta, C = compact myocardium, LA = left atrium, LV = left ventricle, RV = right ventricle.

HiP-CT captured the microanatomy of both the control and diseased hearts at high spatial resolution (see Table S2, Figs 1–6), demonstrating the feasibility of quantitative measurement (Figs 2, 3, S4).

### Structural Anatomy of the Control Heart with Approximately 20-µm Voxel Size

In the control heart, HiP-CT was able to depict subtle differences in density in all important tissue types with approximately 20-µm voxel size (Table S2), with excellent contrast-to-noise ratio (Table). Anatomic boundaries were clear and distinct, facilitating straightforward 3D rendering, segmentation, and quantification (Figs 1, 2, S4; Movie 2). As an example of multiplanar quantification at this resolution, both the presence and degree of mitral annular disjunction (displacement of the mural leaflet of the mitral valve from the ventricular myocardium) and its distribution around the mural aspect of the left atrioventricular (AV) junction were measured and mapped (Fig S4A–S4D). The feasibility of mapping the orientation of myocytic aggregates within the compact myocardium using structure tensor analysis (myomapping) was confirmed, and myocyte orientation demonstrated the expected gradual transition in helical angle from epicardium to endocardium (Fig 3).

**Movie 1:** Control heart, rendered with Siemens Healthineers cinematic rendering.

**Movie 2:** Diseased heart, rendered with Siemens Healthineers cinematic rendering.

### High-Spatial-Resolution Zoom Scans with Approximately 2.3–6.4-µm Voxel Size

In regions of interest viewed with higher spatial resolution, clinically relevant components could be traced in 3D and quantified. Measurement of the transmural thickness of the endo-, myo-, and epicardial layers of the left atrium—relevant to cryoablation for atrial fibrillation—highlighted significant differences in endo- and epicardial thickness of the left and right atria. Specifically, endocardial thickness was significantly greater (*P* < .001) in the left atrium than the right atrium in both hearts. Meanwhile, epicardial thickness was significantly greater (*P* < .001) in the diseased heart than the control heart for both the left and right atria (Fig 2).

HiP-CT was able to demonstrate details of the cardiac conduction system and its interconnectivity with surrounding structures. The sinus node was identified in cross-section as a distinct condensation of tightly packed cells containing small, dense, fibrous elements, surrounding the sinus nodal artery (Fig 4A). Multiple connections between the node and the tightly adjacent atrial myocardium and paranodal area could be traced throughout its length. The compact AV node was located by tracking its artery within the inferior pyramidal space until its first branch point (Fig 4B). Again, the compact node was tightly adjacent to the (vestibular) myocardium of the right atrium. Connections between the AV node and the atrial septum (potential fast pathway) and to the right and left vestibules on either side of the inferior pyramidal space could be traced through virtual multiplanar sectioning (Fig 4B, 4C). Likewise, multiple connections between the AV node and the underlying crest of the ventricular septum could be determined (potential nodoventricular pathways). Of interest, each pathway followed the course of microvasculature, which was accompanied by tissue of an AV nodal phenotype as the microvasculature broke through the fibrous AV insulating tissue (Fig 4C). The course of the penetrating bundle of His, and the proximal left and right bundle branches, could also be traced (Fig S5).

### Structural Anatomy of the Diseased Heart

In the diseased heart, multiple abnormalities in microstructure were visible with approximately 20-µm voxel resolution, with confirmation in high-resolution zoom scans (Table S2). These abnormalities included an increased distance of coronary vasculature from the epicardial surface (Figs 1, 5A), coronary stent with associated calcification (Fig 5B), atherosclerotic plaque with associated calcification (Fig 5C, 5D), lipomatous hypertrophy of the atrial septum and a thrombus in transit through a patent foramen ovale (Fig S6), calcification of the mitral annulus and aortic valvar leaflets (Figs 6, S4), and changes in myocyte structure related to myocardial infarction (Fig 6). Quantitatively, the epicardium of both atria was thicker (Fig 2C) and there was a greater degree and extent of mitral annular disjunction (Fig S4B, S4D) in the diseased heart compared with the control heart. The structure of the sinus node and the AV node was disrupted by fatty infiltration (Fig 4D–4F); the connections between a more stellate sinus node and the surrounding atrial myocardium plus paranodal area were attenuated (Fig 4D), as were the connections between the AV node and the adjacent atrial septum and atrial vestibules (potential disruption of both fast and slow pathways) (Fig 4E, 4F).

## Discussion

In this study, we showed the utility of hierarchical phase-contrast tomography (HiP-CT) for high-spatial-resolution imaging of intact structurally normal and diseased adult human hearts without the need for destructive methodology, sectioning, or exogenous contrast agents. HiP-CT provided multidimensional and histologic-level detail of the myocardium, valves, coronary arteries, and components of the cardiac conduction system across length scales. It provided resolutions ranging from gross (isotropic voxels of approximately 20-µm) to microscopic (approximately 6.4-µm voxel size) to cellular (approximately 2.3-µm voxel size) in scale. It adds to the growing set of x-ray phase-contrast imaging techniques and analyses being applied to biologic systems (25,26).

Multiple challenges and controversies still exist in the field of cardiovascular science and anatomy that HiP-CT has the potential to help resolve. Of note, we have shown that comprehensive analysis of the cardiac conduction system is technically feasible using HiP-CT, thus paving the way for more accurate interpretation of the anatomy underlying arrhythmia as well as strategies for treatment via ablation (27), while also keeping the conduction system in the context of whole-heart anatomy. For example, while indicators of atrial myocyte orientation—relevant to conduction between the sinus and AV nodes—can be visualized using other techniques (13), tracing normal and abnormal pathways between the nodes and underlying working myocardium at the histologic level has proven to be a difficult task, and is usually performed using destructive tissue sectioning (28). As a starting point, our analysis shows the potential to track such pathways in 3D using high-spatial-resolution HiP-CT data and shows that the connections surrounding the sinus node are likely multiple and are present, but attenuated, in the setting of lipomatous hypertrophy. Likewise, we were able to track the course of the AV node in our data sets and show its close proximity to the adjacent myocardium in the control heart. This feature, along with potential connections to the atrial septum (potential fast pathway), was disrupted by fatty infiltration in the diseased heart. Given the well-known association between obesity and arrhythmia (29), these findings are relevant not only for understanding the onset of cardiac rhythm abnormalities but also for the efficacy of ablation strategies to cure them (30). Furthermore, connections between the AV node and ventricular myocardium (potential nodoventricular connections) or between the nonbranching bundle of His or bundle branches and the ventricular septum (fasciculoventricular connections) are notoriously challenging to document histologically, not least because sections are often lost or omitted, even with expert serial sectioning. Again, we were able to trace these potential connections in an uninterrupted manner in our data sets. Of note, we demonstrated the presence of nodoventricular connections in both the healthy and diseased heart.

Besides anatomy related to the cardiac conduction system, we identified three further anatomic features with the potential to be investigated through quantitative analysis of HiP-CT data. First, we showed the feasibility of measuring atrial wall thickness (Fig 2), including the thickness of epicardial fat, which may be relevant to transmural cryoablation for atrial fibrillation. Second, measurement of the orientation of aggregates of myocytes was possible within an intact ventricular mass (Fig 3). Continuing controversies exist in understanding the mural architecture of the compact myocardium; myomapping with the high spatial resolution of HiP-CT could not only aid in more accurate mathematical modeling of myocardial structure in health and disease but also help validate clinically relevant advanced imaging techniques, such as diffusion-tensor MRI. Third, we showed the feasibility of using HiP-CT to investigate mitral annular disjunction (Fig S4), another clinically relevant yet controversial feature of anatomy. Of interest, mitral annular disjunction was demonstrated to be present in both the control and the diseased hearts but was more prominent in the diseased heart, which was from a patient who had both mitral regurgitation and atrial fibrillation.

We propose that HiP-CT, in bridging the gap between the whole-organ and cellular scales, represents a step change in radiologic techniques for ex vivo investigation of the heart. The potential to resolve broad-based anatomic questions relevant to the understanding and treatment of cardiovascular diseases makes HiP-CT a potentially powerful, nondestructive research tool. It could be useful not only for understanding acquired heart disease but also for prospective application in Biobanks of rare and complex congenital heart malformations.

Our study has several limitations. First, HiP-CT requires specialized equipment and expertise. Access to synchrotron radiation facilities worldwide is limited and expensive, which may hinder imaging the larger number of samples required for robust statistical comparison in preclinical series. Second, imaging was performed ex vivo and may not fully reflect the dynamic and muscular behavior of hearts in vivo. In vivo scanning using HiP-CT is currently limited by the high radiation dose associated with synchrotron imaging. Third, sample preservation and preparation, if not optimized, may induce morphologic deformations. In particular, maintaining full patency of vessels and chambers during standard whole-organ anatomic embalming, as used in our study, is challenging and can make segmentation and 3D reconstruction more difficult. Fourth, we did not have histopathologic evaluation to use as a reference standard, and the conclusions reached by studying only two hearts are inherently limited and may not be generalizable.

In conclusion, for ex vivo and virtual autopsy cardiac imaging, this study demonstrates the potential of hierarchical phase-contrast tomography (HiP-CT) to provide high-spatial-resolution, three-dimensional images of structurally normal and diseased adult human hearts. HiP-CT was used to obtain whole-heart image volumes with isotropic voxels of approximately 20 µm and local regions of interest with voxel size down to 2.3–6.4 µm without the need for tissue sectioning, destructive techniques, or exogenous contrast agents. The multiscale approach enables insights into cardiac macro- and microstructure. Because of its potential to provide a comprehensive view of cardiac anatomy and its alterations in disease, HiP-CT represents a promising tool for developing innovative diagnostic and treatment strategies for cardiovascular disease.

## References

[1] Garcia-Canadilla P , Dejea H , Bonnin A , et al . Complex congenital heart disease associated with disordered myocardial architecture in a midtrimester human fetus . Circ Cardiovasc Imaging 2018 ; 11 ( 10 ): e007753 . 30354476 10.1161/CIRCIMAGING.118.007753

[2] Dejea H , Garcia-Canadilla P , Cook AC , et al . Comprehensive analysis of animal models of cardiovascular disease using multiscale x-ray phase contrast tomography . Sci Rep 2019 ; 9 ( 1 ): 6996 . [Published correction appears in Sci Rep 2019;9(1):18278.] 31061429 10.1038/s41598-019-43407-z PMC6502928

[3] Gilbert SH , Benoist D , Benson AP , et al . Visualization and quantification of whole rat heart laminar structure using high-spatial resolution contrast-enhanced MRI . Am J Physiol Heart Circ Physiol 2012 ; 302 ( 1 ): H287 – H298 . 22021329 10.1152/ajpheart.00824.2011 PMC3334235

[4] Lee L , Genge CE , Cua M , et al . Functional assessment of cardiac responses of adult zebrafish (Danio rerio) to acute and chronic temperature change using high-resolution echocardiography . PLoS One 2016 ; 11 ( 1 ): e0145163 . [Published correction appears in PLoS One 2016;11(2):e0149741.] 26730947 10.1371/journal.pone.0145163 PMC4701665

[5] Lee L , Cui JZ , Cua M , et al . Aortic and cardiac structure and function using high-resolution echocardiography and optical coherence tomography in a mouse model of Marfan syndrome . PLoS One 2016 ; 11 ( 11 ): e0164778 . 27824871 10.1371/journal.pone.0164778 PMC5100915

[6] Pichat J , Iglesias JE , Yousry T , Ourselin S , Modat M . A survey of methods for 3D histology reconstruction . Med Image Anal 2018 ; 46 : 73 – 105 . 29502034 10.1016/j.media.2018.02.004

[7] Mai H , Rong Z , Zhao S , et al . Scalable tissue labeling and clearing of intact human organs . Nat Protoc 2022 ; 17 ( 10 ): 2188 – 2215 . 35859136 10.1038/s41596-022-00712-8

[8] Zhao S , Todorov MI , Cai R , et al . Cellular and molecular probing of intact human organs . Cell 2020 ; 180 ( 4 ): 796 – 812.e19 . 32059778 10.1016/j.cell.2020.01.030 PMC7557154

[9] Lombardi CM , Zambelli V , Botta G , et al . Postmortem microcomputed tomography (micro-CT) of small fetuses and hearts . Ultrasound Obstet Gynecol 2014 ; 44 ( 5 ): 600 – 609 . 24585450 10.1002/uog.13330

[10] Reichardt M , Töpperwien M , Khan A , Alves F , Salditt T . Fiber orientation in a whole mouse heart reconstructed by laboratory phase-contrast micro-CT . J Med Imaging (Bellingham) 2020 ; 7 ( 2 ): 023501 . 32206684 10.1117/1.JMI.7.2.023501 PMC7055497

[11] Walton LA , Bradley RS , Withers PJ , et al . Morphological characterisation of unstained and intact tissue micro-architecture by x-ray computed micro- and nano-tomography . Sci Rep 2015 ; 5 ( 1 ): 10074 . 25975937 10.1038/srep10074 PMC4650804

[12] Dejea H , Bonnin A , Cook AC , Garcia-Canadilla P . Cardiac multi-scale investigation of the right and left ventricle *ex vivo*: a review . Cardiovasc Diagn Ther 2020 ; 10 ( 5 ): 1701 – 1717 . 33224784 10.21037/cdt-20-269 PMC7666925

[13] Pashakhanloo F , Herzka DA , Ashikaga H , et al . Myofiber architecture of the human atria as revealed by submillimeter diffusion tensor imaging . Circ Arrhythm Electrophysiol 2016 ; 9 ( 4 ): e004133 . 27071829 10.1161/CIRCEP.116.004133 PMC7035884

[14] Walsh CL , Tafforeau P , Wagner WL , et al . Imaging intact human organs with local resolution of cellular structures using hierarchical phase-contrast tomography . Nat Methods 2021 ; 18 ( 12 ): 1532 – 1541 . 34737453 10.1038/s41592-021-01317-x PMC8648561

[15] Chapman HN . Fourth-generation light sources . IUCrJ 2023 ; 10 ( Pt 3 ): 246 – 247 . 10.1107/S2052252523003585 PMC10161768 37144816

[16] Paganin D , Mayo SC , Gureyev TE , Miller PR , Wilkins SW . Simultaneous phase and amplitude extraction from a single defocused image of a homogeneous object . J Microsc 2002 ; 206 ( Pt 1 ): 33 – 40 . 12000561 10.1046/j.1365-2818.2002.01010.x

[17] Mirone A , Brun E , Gouillart E , Tafforeau P , Kieffer J . The PyHST2 hybrid distributed code for high speed tomographic reconstruction with iterative reconstruction and a priori knowledge capabilities . Nucl Instrum Methods Phys Res B 2014 ; 324 : 41 – 48 .

[18] Lyckegaard A , Johnson G , Tafforeau P . Correction of ring artifacts in X-ray tomographic images . Int J Tomogr Stat 2011 ; 18 ( F11 ): 1 – 9 .

[19] Xian RP , Walsh CL , Verleden SE , et al . A multiscale X-ray phase-contrast tomography dataset of a whole human left lung . Sci Data 2022 ; 9 ( 1 ): 264 . 35654864 10.1038/s41597-022-01353-y PMC9163096

[20] Brunet J , Walsh CL , Wagner WL , et al . Preparation of large biological samples for high-resolution, hierarchical, synchrotron phase-contrast tomography with multimodal imaging compatibility . Nat Protoc 2023 ; 18 ( 5 ): 1441 – 1461 . 36859614 10.1038/s41596-023-00804-z

[21] Maitin-Shepard J , Baden A . Neuroglancer. GitHub . https://github.com/google/neuroglancer. Published 2021. Accessed May 15, 2024 .

[22] Engel K . Real-time Monte-Carlo path tracing of medical volume data . Presented at the 2016 GPU Technology Conference , San Jose, Calif , April 4–7, 2016 .

[23] Schindelin J , Arganda-Carreras I , Frise E , et al . Fiji: an open-source platform for biological-image analysis . Nat Methods 2012 ; 9 ( 7 ): 676 – 682 . 22743772 10.1038/nmeth.2019 PMC3855844

[24] Chatzaraki V , Kubik-Huch RA , Thali M , Niemann T . Quantifying image quality in chest computed tomography angiography: evaluation of different contrast-to-noise ratio measurement methods . Acta Radiol 2022 ; 63 ( 10 ): 1353 – 1362 . 34647842 10.1177/02841851211041813

[25] Barbone GE , Bravin A , Mittone A , et al . High-spatial-resolution three-dimensional imaging of human spinal cord and column anatomy with postmortem x-ray phase-contrast micro-CT . Radiology 2021 ; 298 ( 1 ): 135 – 146 . 33107800 10.1148/radiol.2020201622

[26] Lv WJ , Zhao XY , Hu DD , Xin XH , Qin LL , Hu CH . Insight into bile duct reaction to obstruction from a three-dimensional perspective using ex vivo phase-contrast CT . Radiology 2021 ; 299 ( 3 ): 597 – 610 . 33876972 10.1148/radiol.2021203967

[27] Anderson RH , Sanchez-Quintana D , Mori S , Cabrera JA , Back Sternick E . Re-evaluation of the structure of the atrioventricular node and its connections with the atrium . Europace 2020 ; 22 ( 5 ): 821 – 830 . 32304217 10.1093/europace/euaa031

[28] Kalyanasundaram A , Li N , Augostini RS , Weiss R , Hummel JD , Fedorov VV . Three-dimensional functional anatomy of the human sinoatrial node for epicardial and endocardial mapping and ablation . Heart Rhythm 2023 ; 20 ( 1 ): 122 – 133 . 36113768 10.1016/j.hrthm.2022.08.039 PMC9897959

[29] Wang TJ , Parise H , Levy D , et al . Obesity and the risk of new-onset atrial fibrillation . JAMA 2004 ; 292 ( 20 ): 2471 – 2477 . 15562125 10.1001/jama.292.20.2471

[30] Pambrun T , Duchateau J , Delgove A , et al . Epicardial course of the septopulmonary bundle: anatomical considerations and clinical implications for roof line completion . Heart Rhythm 2021 ; 18 ( 3 ): 349 – 357 . 33188900 10.1016/j.hrthm.2020.11.008

